# Epidermolysis Bullosa Accompanied by Duodenal Atresia in an Infant: A Report of a Rare Case

**DOI:** 10.7759/cureus.77350

**Published:** 2025-01-12

**Authors:** Yaser M Almonla, Shamma Alothman, Salim M Abduljawad, Zakaria S Habib

**Affiliations:** 1 College of Medicine, Alfaisal University, Riyadh, SAU; 2 Surgery, Kingdom Hospital, Riyadh, SAU; 3 Pediatric Medicine, University of Aleppo, Aleppo, SYR

**Keywords:** double bubble, duodenal atresia, epidermolysis bullosa, genetic diseases, multidisciplinary approach, neonatal surgery

## Abstract

Epidermolysis bullosa (EB) is a group of inherited disorders characterized by skin fragility, resulting in blisters and erosions from minor trauma. Duodenal atresia (DA) is a rare congenital malformation that causes luminal obstruction, typically presenting with non-bilious vomiting and abdominal distension in neonates - hallmark signs of proximal intestinal obstruction. The co-occurrence of EB and DA is exceptionally rare and poses unique diagnostic and treatment challenges. We report the case of a term neonate born with widespread skin blistering and erosions, subsequently diagnosed with EB. By the second day of life, the neonate developed non-bilious vomiting and abdominal distension, leading to a diagnosis of DA confirmed radiologically by the characteristic “double bubble” sign. Surgical correction via duodenoduodenostomy was performed, complemented by interdisciplinary care involving wound and pain management as well as nutritional support. Post-surgery, the neonate showed significant improvement, underscoring the importance of early diagnosis and a multidisciplinary approach in managing these complex conditions. However, the long-term prognosis remains guarded due to the chronic nature of EB.

## Introduction

Epidermolysis bullosa (EB) is one of the rarest genetic disorders globally, with an estimated incidence of 1 in 50,000 live births. EB primarily affects the skin, causing fragile skin that blisters and peels easily even with minor trauma. There are three main types of EB: simplex, junctional, and dystrophic, each differing in severity and impact on the skin’s integrity and function [1]. All types can result in significant complications, including scarring, frequent infections, and irreversible damage to healthy skin.

Duodenal atresia (DA) is a congenital condition in which the duodenum is completely obstructed, preventing the passage of food to the distal gastrointestinal tract. This occurs due to the failure of the duodenum to recanalize during embryonic development. DA is often associated with other congenital anomalies, including trisomy 21, cardiac defects, and intestinal malrotation. Its hallmark radiological feature, the “double bubble” sign, shows dilation of the stomach and proximal duodenum caused by an obstruction in the distal segment [2,3].

The co-occurrence of EB and DA is extremely rare, with only a few cases reported in the literature. This combination presents unique diagnostic and therapeutic challenges, particularly in neonates who require immediate and specialized care [4]. A case report of concurrent EB and DA highlights the clinical presentation, diagnostic process, and surgical and medical management strategies required. It also underscores the importance of a multidisciplinary approach to optimize outcomes in managing these complex and rare conditions.

## Case presentation

A healthy 28-year-old mother had an uncomplicated pregnancy and delivered a male neonate preterm at 36 weeks gestation via spontaneous vaginal delivery. The family history was unremarkable for dermatological or congenital anomalies, and there was no consanguinity. The infant had a birth weight of 1,550 grams. The Apgar scores were 8 and 9 at one and five minutes, respectively.

**Figure 1:**
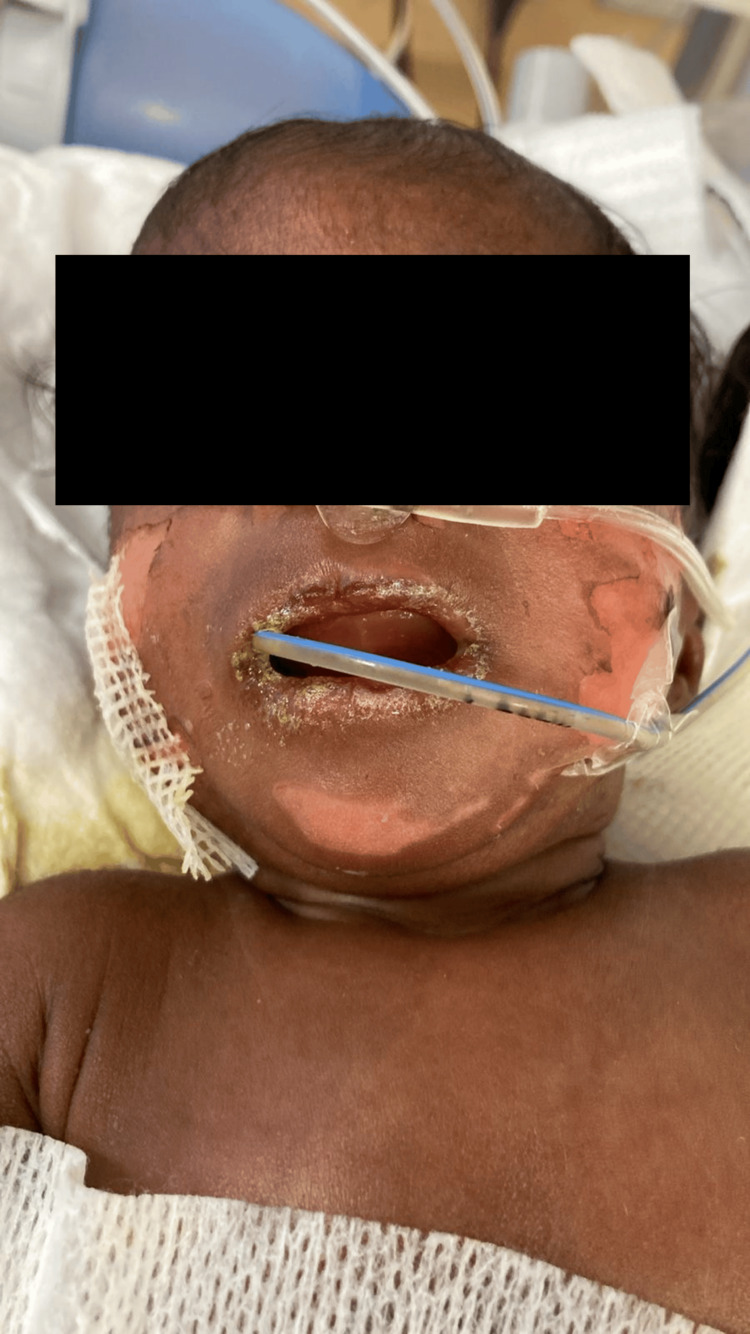
An infant diagnosed with EB EB, epidermolysis bullosa

The infant developed multiple skin blisters and erosions, predominantly on the hands, feet, and areas subjected to mechanical friction, appearing within hours of birth and recurring at various intervals thereafter. The lesions were accompanied by redness and tenderness. The skin was so fragile that new blistering occurred with even minimal handling during routine care. The diagnosis of EB was confirmed by the clinical presentation (Figure 1).

**Figure 2:**
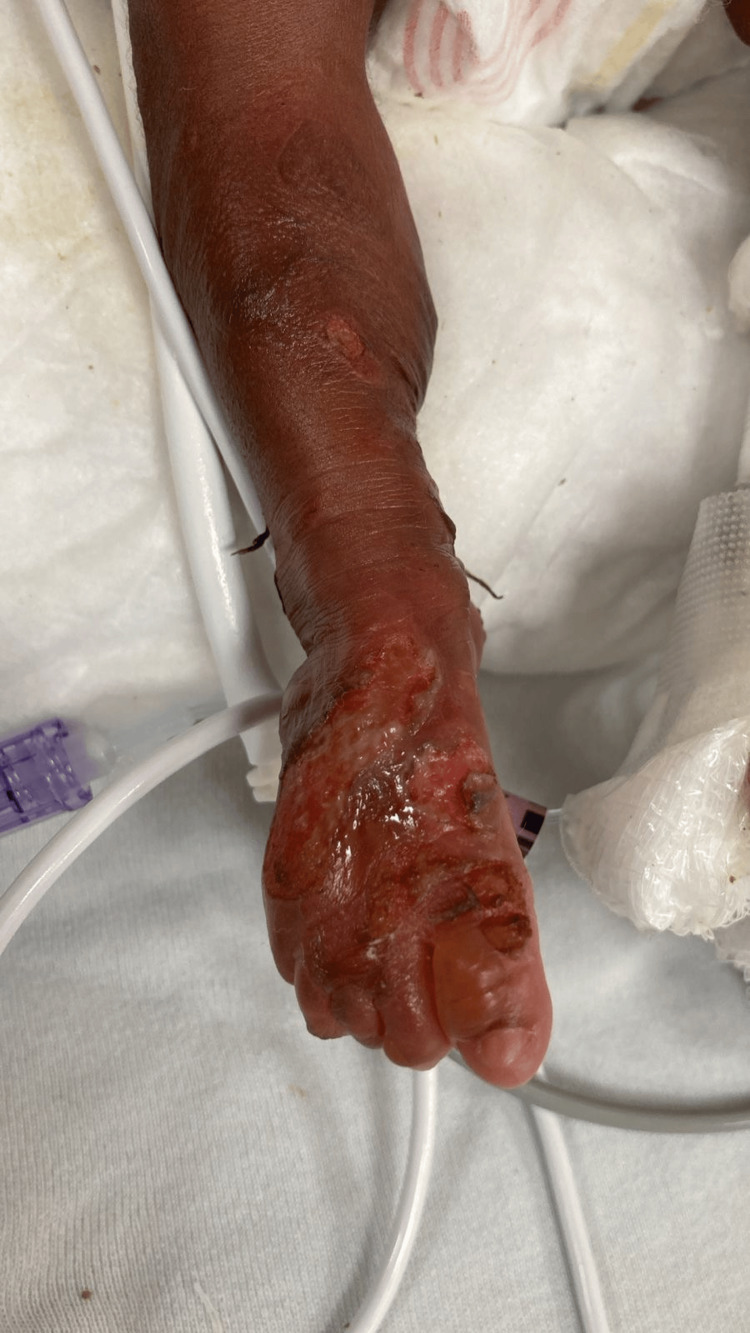
Skin fragility and blister formation

On the second day of life, the infant began experiencing non-bilious vomiting and progressive abdominal distension. Feeding attempts were unsuccessful, and gastric aspirates were significant despite the placement of a nasogastric tube. Physical examination revealed a distended abdomen with visible gastric peristalsis, but no palpable mass. Plain abdominal X-rays showed the characteristic “double bubble” sign, confirming the diagnosis of DA. The neonate was transferred to a tertiary NICU for further evaluation and management. Genetic testing for EB was initiated, with results pending at the time of this report. The severity of skin involvement suggested either junctional or dystrophic EB based on clinical features (Figure 2).

**Figure 3:**
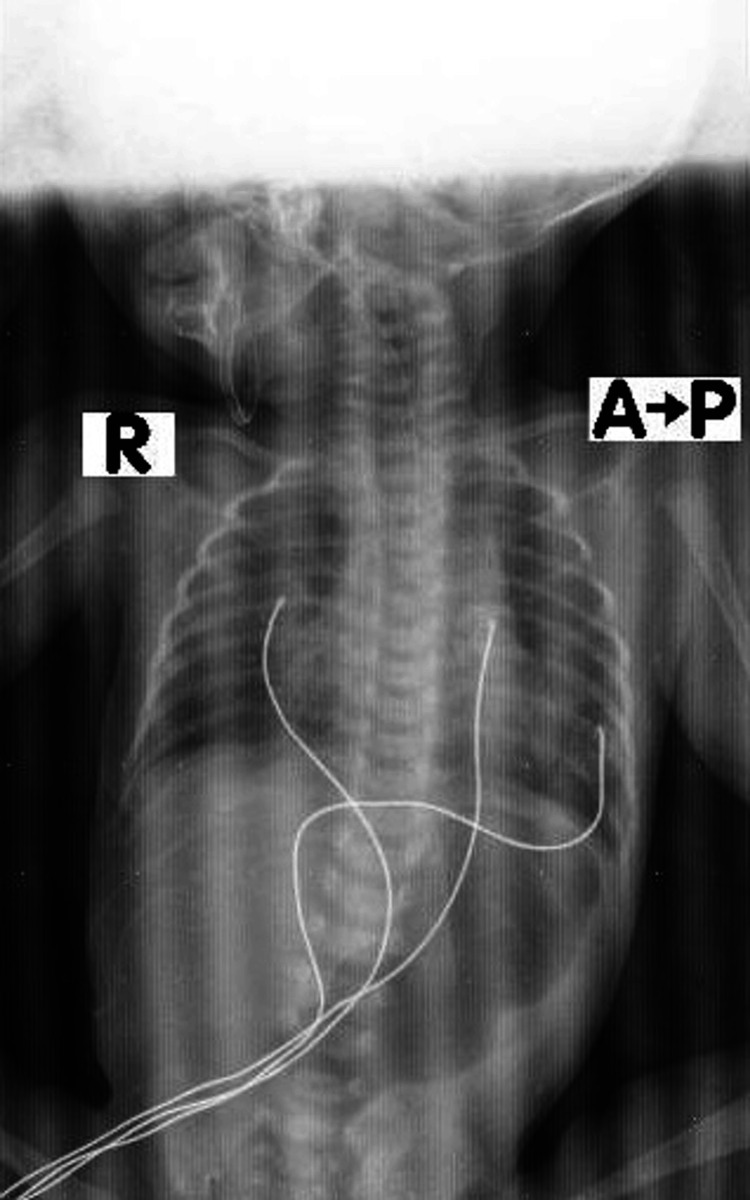
Radiographic representation of DA DA, duodenal atresia

DA was confirmed radiologically. A plain abdominal X-ray revealed the characteristic “double bubble” sign, with no gas distal to the duodenum (Figure 3). This finding is diagnostic of DA, and no further imaging was required. The clinical presentation, including persistent non-bilious vomiting and failure to tolerate feeds, further supported the diagnosis [4].

Management

The case was managed through a multidisciplinary approach involving neonatologists, pediatric surgeons, dermatologists, geneticists, and dietitians. This team collaborated to address the systemic implications of both disorders. Neonatologists oversaw overall neonatal care, pediatric surgeons performed the surgical correction of DA, dermatologists managed the skin fragility associated with EB, geneticists provided diagnostic support and counseling, and dietitians ensured appropriate nutritional support during recovery [3].

**Figure 4:**
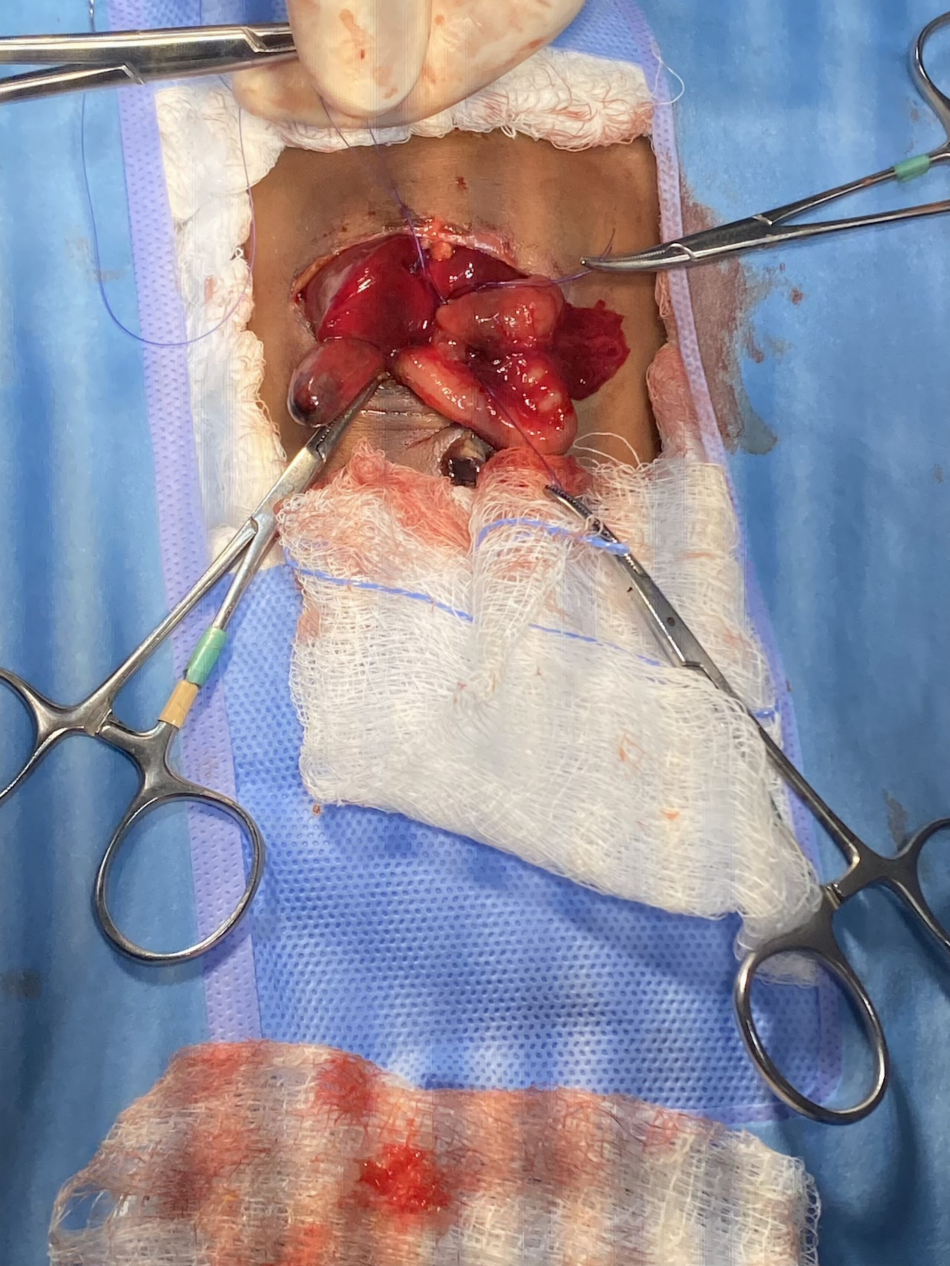
Surgical treatment of DA DA, duodenal atresia

Surgical correction of DA was performed on the second day of life. The neonate underwent duodenoduodenostomy, in which the obstructed segment of the duodenum was bypassed by creating a direct “Kimura” anastomosis between the proximal and distal duodenal segments (Figure 4). Extra precautions were taken to ensure the skin was not injured during preoperative preparations and surgical positioning. The surgery was uneventful, with no postoperative emergencies. The neonate was maintained on nil per os (NPO) status while receiving parenteral nutrition.

Management of EB focused on minimizing tissue damage and providing optimal skin care to promote healing. Handling and dressing changes were performed carefully to avoid new blister formation. Emollient-soaked, non-adherent dressings were applied to the affected areas, and the dressings were not changed frequently to minimize further skin damage. The environment was adjusted to prevent friction by placing soft pads and fabrics on all contact surfaces.

Pain management was a critical concern due to the pain associated with handling and wound care. The neonate was treated with acetaminophen as a first-line analgesic, and low-dose opioids, including fentanyl, were administered. For the dressing change procedure, morphine in injectable form was used. On postoperative day one, following a period of NPO with parenteral nutrition, the neonate was transitioned to oral nutritional supplements. This approach provided adequate calories to support wound healing and recovery after surgery. Initially, parenteral nutrition was given, followed by enteral feedings with a hydrolyzed formula to reduce gastrointestinal stress. The neonate’s nutritional status was closely monitored, and adjustments were made based on growth and metabolic patterns.

## Discussion

EB is a genetically and clinically diverse skin disorder characterized by skin blistering of varying severity. The probable subtypes in this case, junctional EB and dystrophic EB, result from genetic mutations that compromise the structural integrity of the dermo-epidermal junction. Specifically, mutations in genes such as LAMA3, LAMB3, and LAMC2 lead to deficient epidermal-dermal adhesion due to defective laminin-332, while mutations in COL7A1, which encodes type VII collagen, cause dystrophic EB through disruption of anchoring fibrils [5]. These genetic insights explain the deep tissue cleavage, recurrent blistering, and the heightened risk of systemic complications, such as infections and chronic pain, seen in these patients. Even basic interventions, such as intravenous line placement or repositioning, can exacerbate blistering, requiring meticulous wound care with non-adherent dressings and emollients to minimize additional trauma. Pain management, often overlooked in neonates, became a critical aspect of care to alleviate the severe discomfort caused by wound dressings and procedures.

DA results from the failure of the duodenum to recanalize during the sixth to eighth weeks of gestation and is often associated with chromosomal abnormalities, such as trisomy 21. Isolated DA remains poorly understood genetically, and its co-occurrence with EB is exceptionally rare, with fewer than 10 cases reported in the medical literature [6,7]. This rarity limits our understanding of any potential genetic or embryological connections between the two conditions. In this case, DA caused feeding intolerance, vomiting, and abdominal distension, which necessitated surgical intervention. The fragile, blistered skin complicated surgical preparation and positioning, requiring sensitive modifications to standard procedures. Successful surgical outcomes demonstrated the importance of a personalized and technical approach to planning and care.

Supplemental feeding was a central focus due to the interplay between DA and EB. Initial parenteral nutrition transitioned to enteral feeding with hydrolyzed formulas following surgical repair to minimize gastrointestinal stress. The increased nutritional demands associated with chronic wound healing in EB, including higher protein turnover and energy requirements, necessitated a tailored nutritional plan to support growth and tissue regeneration [6,8].

EB and DA both present significant challenges in neonatal care due to their rarity, but when co-occurring, their combined impact on newborns increases the complexity of care. The coexistence of these conditions requires close collaboration between multiple disciplines [9,10]. This case highlights the critical role of a multidisciplinary team - comprising neonatologists, pediatric surgeons, dermatologists, geneticists, and dietitians - in addressing the complex needs of infants with both EB and DA [10]. The low prevalence of their co-occurrence underscores the need for personalized treatment plans and offers opportunities for further research into potential genetic or embryological connections. Genetic insights, particularly concerning mutations in LAMA3, LAMB3, LAMC2, and COL7A1, may help clarify subtypes and guide future management strategies. Further studies of such rare and complex cases are essential to improving patient outcomes. This case underscores the importance of individualized care and a collaborative approach in managing the rare coexistence of EB and DA.

## Conclusions

EB with DA is a rare and complex condition that necessitates a coordinated, interdisciplinary approach. Managing such cases requires specialized microsurgery for DA, comprehensive wound care, effective pain management, and tailored nutritional therapy for EB. Collaboration among neonatologists, pediatric surgeons, dermatologists, geneticists, and dietitians is essential to ensure optimal care. Further research is needed to explore potential genetic or embryological links between these conditions and to enhance management strategies for such rare cases.
